# Portable and wearable self-powered systems based on emerging energy harvesting technology

**DOI:** 10.1038/s41378-021-00248-z

**Published:** 2021-03-17

**Authors:** Chen Xu, Yu Song, Mengdi Han, Haixia Zhang

**Affiliations:** 1 Academy for Advanced Interdisciplinary Studies, Peking University, Beijing, China; 2 National Key Laboratory of Science and Technology on Micro/Nano Fabrication, Institute of Microelectronics, Peking University, Beijing, China; 3 Department of Biomedical Engineering, College of Future Technology, Peking University, Beijing, China

**Keywords:** Nanoscale devices, NEMS

## Abstract

A self-powered system based on energy harvesting technology can be a potential candidate for solving the problem of supplying power to electronic devices. In this review, we focus on portable and wearable self-powered systems, starting with typical energy harvesting technology, and introduce portable and wearable self-powered systems with sensing functions. In addition, we demonstrate the potential of self-powered systems in actuation functions and the development of self-powered systems toward intelligent functions under the support of information processing and artificial intelligence technologies.

## Introduction

In recent years, portable and wearable electronic devices have been in a stage of rapid development^1,2^. Personalized electronic devices such as smart watches and smart glasses have sprung up, bringing much convenience to people’s life^3,4^. At the same time, with the promotion of flexible electronic technology^5^, big data technology^6,7^ and artificial intelligence technology^8^, portable and wearable electronic devices have shown the development trend of flexibility, integration, and intellectualization, which have also facilitated rich applications such as health monitoring^9,10^, human–machine interaction^11,12^, and the Internet of Things^13,14^.

For portable and wearable electronic devices, the energy supply is a major obstacle to its flexible and integrated application. Replaceable batteries are now the common energy source of electronic devices. However, the rigid characteristics of these batteries limit the overall flexibility of electronic devices. The limited life of batteries and potential environmental pollution problems also do not conform to the principles of sustainable development. As a result, many efforts have been made to explore new environmentally friendly, renewable energy sources to power electronic devices.

Self-powered technology provides a solution for the sustainable energy supply of portable and wearable systems. Self-powered technology means that the device can maintain its own operation by collecting energy in the working environment without an external energy supply. The effective collection of various forms of energy in the working environment is the basis of self-powered technology.

The energy sources available for portable and wearable electronic devices, such as mechanical energy, thermal energy, chemical energy, and solar energy, are extensive. According to the characteristics of these forms of energy, energy harvesting systems suitable for collecting various forms of energy have gained substantial attention. For example, triboelectricity is generated during the contact-separation process of two different materials. Piezoelectricity is produced during the mechanical deformation of piezoelectric materials, and electromagnetic power comes from the conductor moving through a stationary magnetic field. Conversion from mechanical energy to electrical energy exists in all the above transduction mechanisms. Therefore, triboelectric^15,16^, piezoelectric^17,18^, and electromagnetic^19,20^ energy harvesters are suitable for harvesting mechanical energy during human motion. Biofuel cells use enzymes or microbes as catalysts to convert chemical energy into electrical energy. The large amount of chemical energy contained in the biochemical environment of the human body can act as an ideal energy source for biofuel cells, making it a potential method for harvesting human chemical energy^21,22^. The human body temperature is constant, and there is a temperature difference with the outside world. Therefore, thermoelectric generators can convert the heat of the human body into usable electric energy as a stable source of energy^23,24^. Solar energy is also a kind of green renewable clean energy that is an ideal power source for wearable electronic devices^25,26^. Furthermore, hybrid energy harvesters that integrate capabilities of harvesting various forms of energy further improve the efficiency of energy harvesting and broaden the application scenarios^27,28^.

Sensing technology plays a decisive role in our life as an important way for electronic devices to obtain external information. With the development of self-powered technology, self-powered sensing systems that can sense important physical^29,30^, chemical^31,32^, and biochemical^33,34^ information without an external energy supply have gradually come into people’s view. Since there are no issues such as battery replacement and environmental pollution, a self-powered sensing system is expected to be the major form of sensor nodes in the future Internet of Things era. There are two main ways to realize self-powered sensing. Active sensing can be realized by using the output electrical signal itself as the sensing signal. For example, a triboelectric signal can be used as a sensing signal to realize pressure sensing^35^. Using energy harvesting technology to provide energy to a sensor module is another way to realize self-powered sensing^36^. For instance, many self-powered systems based on piezoelectric^37^, thermoelectric^38^, wet electricity^39^, and redox electricity^40^ have been studied to power sensor modules and realize active sensing.

Electronic devices such as actuators can assist humans in completing diverse and complex operations in specific scenarios. The development of self-powered technology makes it possible to realize various actuation functions without an external energy supply. For example, many researchers use electrical energy converted from other forms of energy as an excitation signal to realize the functions of automatic control^41,42^, microfluidics^43,44^, drug delivery and release^45^, and adjuvant therapy^46,47^.

From a long-term point of view, we will eventually witness human society entering the age of intelligence. The Internet of Things, artificial intelligence, and big data technology change our lives with each passing day. The relationship between human beings and electronic devices has also presented an unprecedented state. Electronic devices with a single function will no longer meet the functional requirements of portable electronic devices in the intelligent era. Portable and wearable self-powered intelligent systems are gradually replacing bulky computers as the interface of a new generation of intelligent human–machine interactions and playing an important role in intelligent identification^48^, intelligent control^49^, and other fields.

Self-powered systems are gradually becoming the mainstream trend in the development of electronic devices. Recently, some articles have summarized the latest development of self-powered systems. For example, Khalid et al. summarized the human-powered energy harvesting technology that can be used in smart electronic systems^50^. Dong et al. started with self-powered sensors and presented state-of-the-art works based on the synergy between wearable sensors and AI technology^51^. Wang et al. and Gunawardhana et al. focused on triboelectric energy harvesting technology and introduced in detail the development and application of wearable triboelectric nanogenerators (TENGs) in self-powered systems^52,53^. However, the integration of multiple energy harvesting methods and the diversification of system functions are inevitable development directions of self-powered systems in the future. Therefore, a comprehensive overview of multiple energy harvesting methods and the diverse applications of self-powered systems helps to further establish the connection between energy harvesting and self-powered applications. Starting from such a foothold, we covered a wide range of energy harvesters and diversified self-powered system functions in this review. We first focus on the development of self-powered systems based on emerging energy harvesting technology. In the second part, portable and wearable energy harvesting technology based on various principles is introduced. The third part presents some self-powered sensing systems based on various principles. The fourth part mainly introduces the representative self-powered actuation system. The fifth part mainly shows the self-powered intelligent system as the human–machine interaction interface, and the synergy between self-powered sensors and AI technology is presented. In the sixth part, we discuss our perspective of the development direction of this field.

## Portable and wearable energy harvester

Energy harvesting is the basis of a self-powered system. Additionally, for consideration of convenience and environmental protection, we need sustainable, clean, and renewable energy to power portable and wearable devices. There are various forms of energy in the environment, including not only the energy produced by the human body itself but also the energy provided by the external environment. In daily life, human mechanical movements such as finger movement, walking, and running can produce considerable mechanical energy. However, due to the multimode and low-frequency characteristics of human mechanical movement, it is not easy to collect the mechanical energy of the human body effectively.

Triboelectric and piezoelectric generators are the two most common ways to collect mechanical energy generated by human motion. Triboelectric energy harvesting is based on the well-known principle of friction electrification. The contact of two different objects will induce static charges on the surface of the objects. Subsequently, the relative motion between the two charged objects will produce a potential difference, thus driving the flow of charges. Due to the advantages of a wide selection of materials, low operating frequency and high output power, TENGs have become the most common ways of collecting the mechanical energy of human motion.

**Fig. 1:**
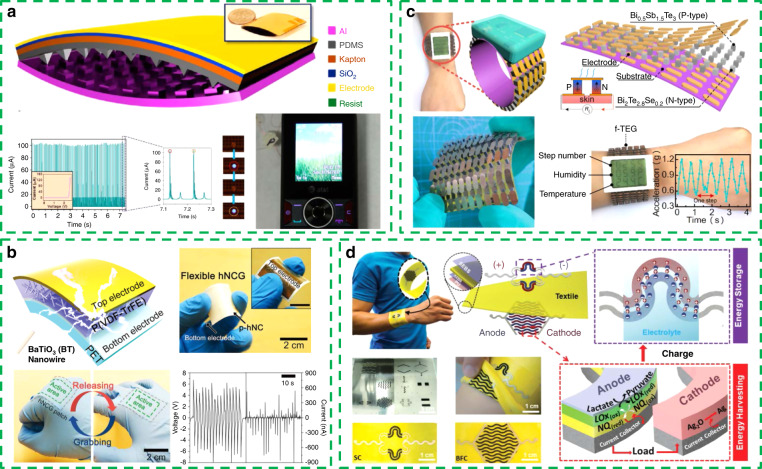
Portable and wearable energy harvester with a single method. **a** Arch-shaped TENG as a power supply for mobile phones. Reprinted from ref. ^54^ with permission. **b** Hybrid nanocomposite generator (hNCG) for hand movement energy harvesting. Reprinted from ref. ^68^ with permission. **c** Flexible thermoelectric generator (f-TEG) for harvesting human thermal energy. Reprinted from ref. ^73^ with permission. **d** Wearable textile-based hybrid supercapacitor–biofuel cell (SC–BFC) system as a biochemical energy harvester. Reprinted from ref. ^77^ with permission.

In Fig. 1a, an arch-shaped TENG was proposed by Z. L. Wang’s research group in 2012^54^. The pyramid patterns on the surface of the TENG help increase the output of the TENG by increasing the contact area of the two triboelectric layers. The output voltage, current density, and energy volume density of the TENG reached 230 V, 15.5 μA cm^−2^ and 128 mW cm^−3^, respectively. The energy conversion efficiency is as high as 10–39% and meets the demands of wireless sensor systems and mobile phones. This work demonstrates for the first time the potential of TENGs for driving personal mobile electronic devices and shows how TENGs can affect lifestyle.

Since invented by Wang in 2012, TENGs have been studied systematically in materials^55,56^, structure^57,58^, working mode^59–62^, and power management^63,64^, during which time, the output of TENGs has been greatly improved. As the most suitable energy harvesting method for human motion mechanical energy, TENGs still face some shortcomings that need to be overcome. For example, stability and reliability are the largest problems. The ability of reliable power management and tolerance to environmental factors such as humidity and temperature also need to be further studied.

As another common means of harvesting mechanical energy, piezoelectric generators have particularly attracted much attention because of their high energy density characteristics and clearer physical mechanism. Piezoelectric energy harvesting is based on the piezoelectric effect. Electrical energy can be generated by the mechanical deformation of piezoelectric materials. Common inorganic piezoelectric materials are hard materials^65^ and are not suitable for portable and wearable devices. With the development of materials science, flexible polymer piezoelectric materials have entered people’s field of vision^66,67^. With excellent flexibility, polymer piezoelectric materials can conformally attach to the surface of the human body, greatly improving wearability and further promoting the development of piezoelectric energy harvesters for human body mechanical energy harvesting.

Jeong et al. developed a 1D–3D (1–3) fully piezoelectric nanocomposite using perovskite BaTiO3 (BT) nanowire-employed poly(vinylidene fluoride-co-trifluoroethylene) (P(VDF-TrFE)) for a high-performance hybrid nanocomposite generator (hNCG) device (Fig. 1b). The output of the flexible hNCG reached 14 V and 4 μA when used for hand movement energy harvesting. Such output performance is higher than the current levels of even previous piezoceramic film-based flexible energy harvesters^68^.

In addition to collecting hand movement energy, piezoelectric energy harvesters also play important roles in intelligent shoes^69^, implantable devices^70^, and intelligent fabrics^71^ for biomechanical energy harvesting. Compared with TENGs, piezoelectric energy harvesters may be limited by the range of material selection. However, the advantages of a higher output current, simple structure, and working mode still make piezoelectric generators an indispensable method for mechanical energy harvesting.

In addition to the mechanical energy produced by human motion, the heat energy of the human body is also valuable energy that can be collected and utilized. The body temperature is constant, and there is a temperature difference with the external environment. Therefore, the use of a thermoelectric generator can achieve continuous energy harvesting. The working principle of a thermoelectric generator is based on the Seebeck effect, that is, the diffusion of electrons and holes caused by a temperature gradient.

There have been many studies on flexible thermoelectric generators (f-TEG) using inorganic thin-film thermoelectric materials or organic compound materials^72^. However, the energy conversion efficiency of these thermoelectric generators is too low to meet the power demand of portable and wearable electronic devices.

To address this problem, Yuan et al. proposed a systematic optimization method for designing a f-TEG (Fig. 1c)^73^. Through optimizing the number of thermoelectric grains, the fill factor and the series–parallel connection mode of the f-TEG, high energy efficiency and power matching with a wearable sensory system are achieved. Such a highly efficient f-TEG utilizing bismuth telluride grains assembled on a flexible polyimide substrate exhibits excellent power performance with power densities of 3.5 µW cm^−2^ and 12.3 µW g^−1^ and can drive the sensor module and finally form a self-powered wearable human body sensing system. Compared with mechanical energy harvesting technology, thermoelectric generators are passive energy harvesting technologies. A thermoelectric generator can achieve continuous and long-term energy collection without any movement of the human body because of the stable energy source. By further increasing the power output, thermoelectric generators may become an ideal energy supply method for portable and wearable self-powered devices in the future.

The human body is a complex physiological environment. In addition to biomechanical energy and heat energy, the human body also has available chemical energy. Biofuel cells are an energy harvesting technology that can collect chemical energy from the human body. Biofuel cells are mainly divided into enzyme-catalyzed biofuel cells and microbial cell catalytic fuel cells that use biofuels such as ethanol^74^ or glucose^75^ to realize the conversion of chemical energy to electric energy.

In the current research, the output power density of biofuel cells is approximately a few microwatts per square centimeter. This level of energy output makes the biofuel cell insufficient to supply sufficient energy in any actual scenario^76^.

Therefore, Lv et al. integrated biofuel cells into self-charging units and presented a wearable textile-based hybrid supercapacitor–biofuel cell (SC–BFC) system (Fig. 1d)^77^. This kind of biofuel cell can scavenge biochemical energy from human sweat and store it in a supercapacitor module. A hybrid energy system integrated with an energy harvesting and energy storage module can solve the problem of the small output energy of biofuel cells and ensure a stable energy supply.

On the basis of single energy harvesting technology, a hybrid energy harvesting system integrated with multiple modes takes advantage of various energy harvesting methods and improves the energy efficiency. For example, biomechanical energy can only be collected when the human body maintains a specific motion posture, while the thermoelectric generator can continuously harvest energy regardless of the human state.

**Fig. 2:**
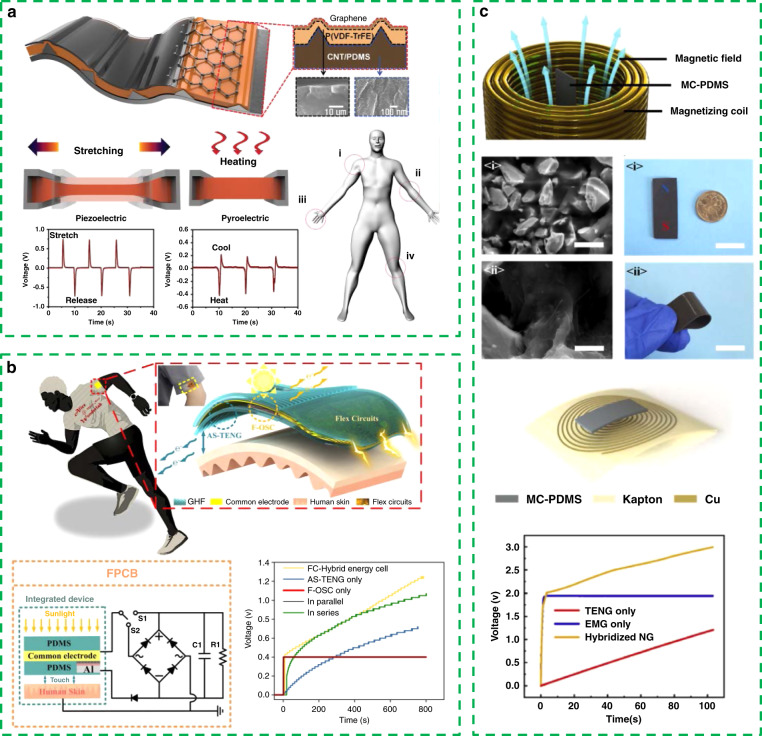
Portable and wearable hybrid energy harvester. **a** Piezoelectric and thermoelectric hybrid energy-scavenging nanogenerator. Reprinted from ref. ^78^ with permission. **b** Solar-triboelectric hybrid energy harvesting system. Reprinted from ref. ^79^ with permission. **c** Hybrid energy harvester based on triboelectric and electromagnetic principles. Reprinted from ref. ^80^ with permission.

In Fig. 2a, Lee et al. fabricated a highly stretchable, hybrid energy-scavenging nanogenerator based on a micropatterned piezoelectric P(VDF-TrFE) polymer, a micropatterned polydimethylsiloxane (PDMS)–carbon nanotube composite and graphene nanosheets^78^. The P(VDF-TrFE) polymer has both a piezoelectric effect and excellent thermoelectric properties with pyroelectric coefficients up to ≈200 µCm^−2^ K^−1^. This kind of hybrid energy harvester can realize the simultaneous collection of biomechanical energy and heat energy when attached to a human hand, a shoulder, an elbow, and other parts.

As a kind of sustainable clean energy, solar energy plays an important role in the field of energy harvesting. The simultaneous collection of solar energy and biomechanical energy is also regarded as an effective means to improve energy collection efficiency. H. X. Zhang’s group proposed a solar-triboelectric hybrid energy harvesting system (Fig. 2b)^79^. Through the design of a common electrode structure and the introduction of an energy management module, this kind of hybrid energy harvester achieves a better charging effect than a single energy harvesting mode in the charging test of capacitors.

In addition, this research group has also proposed a hybrid energy harvester based on triboelectric and electromagnetic principles (Fig. 2c)^80^. Compared with TENGs, electromagnetic generators are different in principle, output characteristics, and applicable frequency. Therefore, integration can achieve complementary advantages to adapt to a variety of applications. In this work, a flexible hybrid energy harvester is proposed based on magnetic and conductive PDMS material, which complements the large output voltage characteristics of TENGs and the large output current characteristics of electromagnetic generators. This hybrid energy harvester can charge a capacitance of 10 μf to 3 V in 110 s, which is superior to the TENG or EMG only.

Energy harvesting technology is the basis of self-powered systems, giving these systems the ability to achieve some functions without an external energy supply. As a necessary way for electronic devices to perceive the external environment, sensors are the cornerstone of the rich functions of electronic devices. Sensors used in wearable scenes can also act as an extension of five human sense organs, giving humans stronger environmental perception capabilities. Therefore, self-powered systems have great application potential in the wearable sensing field.

## Portable and wearable self-powered sensing system

On the basis of energy harvesting technology, a variety of portable, wearable self-powered sensors for monitoring physical, chemical, and physiological information have been developed. There are two main ways to realize self-powered sensing. The first is active sensing, which uses the electrical signal itself as the sensing signal, where the output electrical signal will be affected by some external factors. Active sensing has been widely used in the monitoring of pressure^81^, humidity^82^, and temperature^83^.

**Fig. 3:**
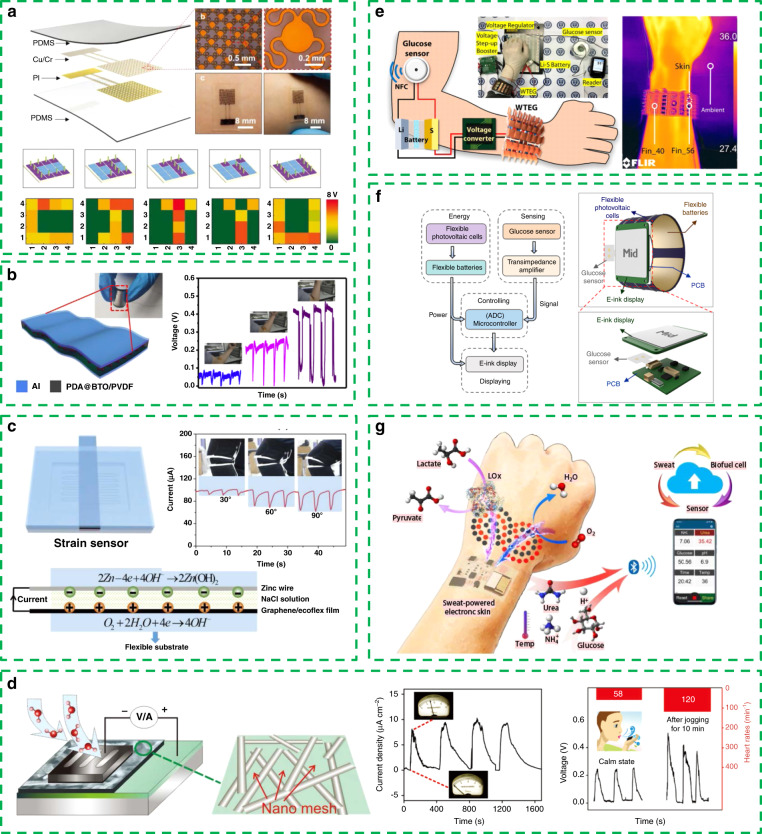
Portable and wearable self-powered sensing system. **a** Self-powered epidermal electronics with the tactile sensing function. Reprinted from ref. ^84^ with permission. **b** Flexible wearable pressure sensor based on piezoelectric materials. Reprinted from ref. ^85^ with permission. **c** The new kind of self-powered strain sensor based on redox electricity. Reprinted from ref. ^86^ with permission. **d** Active humidity sensor based on a moisture-driven electrical generator. Reprinted from ref. ^87^ with permission. **e** Self-powered thermoelectric glucose sensing system integrating the wearable thermoelectric generator (WTEG). Reprinted from ref. ^88^ with permission. **f** Self-powered glucose sensing smart watch based on photovoltaic cells. Reprinted from ref. ^89^ with permission. **g** Flexible perspiration-powered electronic skin (PPES). Reprinted from ref. ^90^ with permission.

In Fig. 3a, Liu et al. reported self-powered epidermal electronics with a tactile sensing function^84^. This kind of epidermal electronics can reflect the pressure on the skin, as well as the pressure distribution, through the triboelectric signal, which is of broad potential interest in wearable electronics.

The output of the piezoelectric material will be affected by its deformation degree, which allows these materials to realize the active sensing of human body postures.

Yang et al. showed a flexible wearable pressure sensor based on piezoelectric materials (Fig. 3b)^85^. On the basis of organic/inorganic piezoelectric material BaTiO3(BTO)/polyvinylidene fluoride(PVDF) composites, polydopamine was introduced as a surface modifier to modify BTO, bringing the improved dispersion of BTO in the organic PVDF matrix. The pressure sensor can realize the active sensing of the elbow bending posture when placed on the elbow of the human body.

In addition, a new self-powered strain sensor based on redox electricity has been proposed using a graphene/Ecoflex film and meandering zinc wire (Fig. 3c)^86^. In the state of stretching, the resistance of graphene/Ecoflex increases with increasing strain, which leads to a decrease in the redox current. This sensor has been utilized to realize the active motion detection of knee joints.

However, pressure sensors based on triboelectric or posture sensors based on piezoelectric and redox electricity are only small-scale laboratory devices as the primary prototype. To achieve a stable wearable active sensor that can cover the whole body of the human body, it is also necessary to solve key problems such as weak device stability and a single sensing mode.

In Fig. 3d, Shen et al. developed an active humidity sensor based on a moisture-driven electrical generator, which realizes the sensing of another physical quantity in addition to the common pressure and strain^87^. The output voltage of the generator is strongly dependent on the humidity of the ambient environment. This new type of device can play the role of self-powered wearable human breathing monitors and touch pads.

In addition to active sensing, using an energy harvester as an energy supply, driving the sensor module is another way to realize wearable self-powered sensing. This mode is mainly used for monitoring physiological indexes of the human body, such as glucose, urea, NH4+, and pH.

In Fig. 3e, Kim et al. proposed a self-powered thermoelectric glucose sensing system integrating a wearable thermoelectric generator (WTEG) and an emerging Li-S battery^88^. WTEG can provide a sufficient power output (378 μW) to drive the commercial glucose sensor and store the remaining energy in the Li-S battery. It can provide a stable energy supply even when the power supply and demand fluctuate greatly. This work demonstrates for the first time the feasibility of fully utilizing human body heat to drive commercial sensors.

Figure 3f presents a self-powered glucose sensing smart watch based on photovoltaic cells^89^. The energy collected by photovoltaic cells can be used to drive the sweat glucose sensor and enable real-time and in situ data analysis/display for driving e-ink screens.

The human body is a complex physiological system. In addition to glucose, the physiological indicators closely related to human health include urea, NH4+, pH, etc. Therefore, it is of great significance for real-time human metabolic health monitoring to realize the self-powered sensing of multiple physiological indicators at the same time.

As shown in Fig. 3g, W. Gao’s research group recently proposed a flexible perspiration-powered electronic skin that uses human sweat to generate electricity^90^. The unique integration of the zero- to three-dimensional nanomaterials helps the device reach a record breaking power density of 3.5 mW cm^−2^ for biofuel cells in untreated human body fluids (human sweat). This electronic skin has the ability to perform multiplexed metabolic sensing in situ and can wirelessly transmit the data to a user interface using Bluetooth technology. Such a self-powered sensor system with a high energy output and a multiple indicator monitoring capability has important application value in human metabolic monitoring, early diagnosis of diseases and other scenarios.

At present, the function of self-powered sensing systems has been greatly enriched. However, facing the obstacles of long-term stability, multimode sensing ability, and energy harvesting efficiency, the self-powered energy system has a long way to go before it can be used in large-scale applications.

## Self-powered actuation system

In addition to wearable sensing functions, self-powered systems can also realize actuations such as object transportation, automatic control, drug delivery and release, adjuvant therapy, and microrobot control. In this section, some actuation applications of self-powered systems are briefly summarized.

**Fig. 4:**
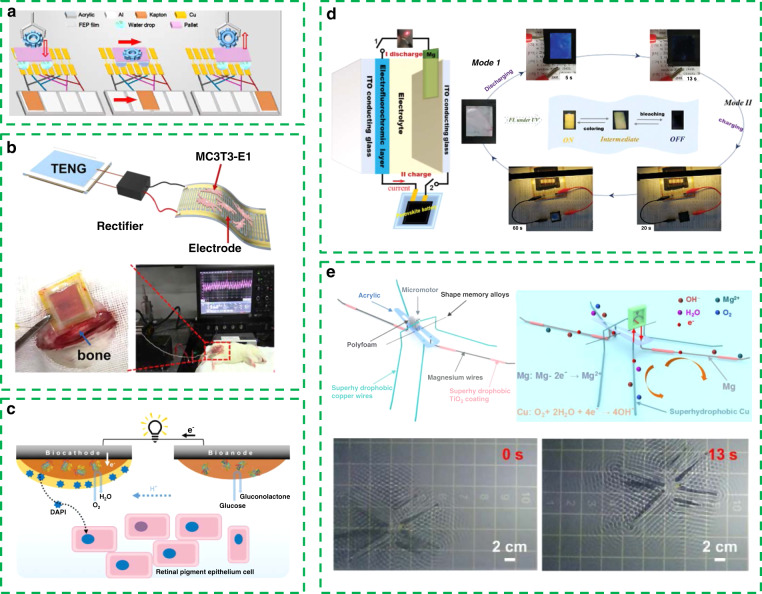
Self-powered actuation system. **a** Self-powered microfluidic transport system combining electrowetting with a TENG. Reprinted from ref. ^91^ with permission. **b** Flexible implantable electrical stimulator composed of a TENG and a flexible interdigital electrode. Reprinted from ref. ^92^ with permission. **c** Self-powered drug delivery system based on enzyme biofuel cells. Reprinted from ref. ^93^ with permission. **d** Self-powered electrofluorochromic devices in intelligent windows. Reprinted from ref. ^94^ with permission. **e** Micro self-powered robot. Reprinted from ref. ^95^ with permission.

Electrowetting refers to changing the wettability of droplets on the substrate by changing the voltage between the droplet and the insulating substrate, that is, changing the contact angle to make the droplets deform and shift. This process is often used to control the position and velocity of a fluid in microfluidic systems. Figure 4a shows a self-powered microfluidic transport system combining electrowetting with a TENG^91^. Here, a miniature vehicle composed of four droplets can transport small objects on the track electrodes when driven by a TENG. Such a self-powered transport system shows great applications in the fields of microsolid/liquid manipulators, drug delivery systems, microrobotics, etc.

In addition, TENGs also play an important role in clinical adjuvant therapy. Tian et al. presented a flexible implantable electrical stimulator composed of a TENG and a flexible interdigital electrode, as shown in Fig. 4b^92^. This electrical stimulation can significantly promote the adhesion, proliferation, and differentiation of osteoblasts and increase the intracellular Ca2+ level. Obviously, this self-powered electrical stimulation system shows great application potential in implantable medical devices and the clinical treatment of osteoporosis and osteoporosis-related fractures.

The transportation and release of drugs without an external power supply is also an important part of intelligent medicine. As shown in Fig. 4c, Xiao et al. proposed a self-powered drug delivery system based on enzyme biofuel cell^93^. Upon discharging the biofuel cell, the drug doped with a conductive polymer will be released rapidly. This work demonstrated the control and ex situ use of ibuprofen, fluorescein, and 4′,6-dimediyl-2-phenylindole (DAPI) and confirmed the feasibility of self-powered technology in drug transport and release scenarios.

Self-powered actuation systems also have important application value in smart homes. Figure 4d shows the application of self-powered electrofluorochromic devices in intelligent windows^94^. Perovskite solar cells can convert solar energy into electrical energy and rapidly color electrochromic materials.

The work in Fig. 4e shows a micro self-powered robot that can walk on the surface of water^95^. Superhydrophobic wires as artificial legs can obtain electric energy from water by redox reactions to supply power to the micromotor and drive the microrobot to move on water. Such a way of driving a robot could provide open prospects for developing self-powered microrobots in aquatic environments.

Self-powered actuation systems play important roles in medical, industrial, and household applications. With the development of processing technology, self-powered actuation systems are expected to undertake more sophisticated and complex functions in the future and further liberate human hands.

## Portable and wearable self-powered smart system

In the era of big data and artificial intelligence technology, electronic devices are no longer limited to a single sensing or actuation function. It is an inevitable trend for portable and wearable electronic devices to develop toward the direction of intelligence^96^. In the future, wearable electronic devices with powerful data processing capabilities will become a new interface of human–machine interaction. In this section, we introduce the application of wearable self-powered systems in human–machine interaction scenarios and then show the synergy of wearable self-powered systems and machine learning technology.

**Fig. 5:**
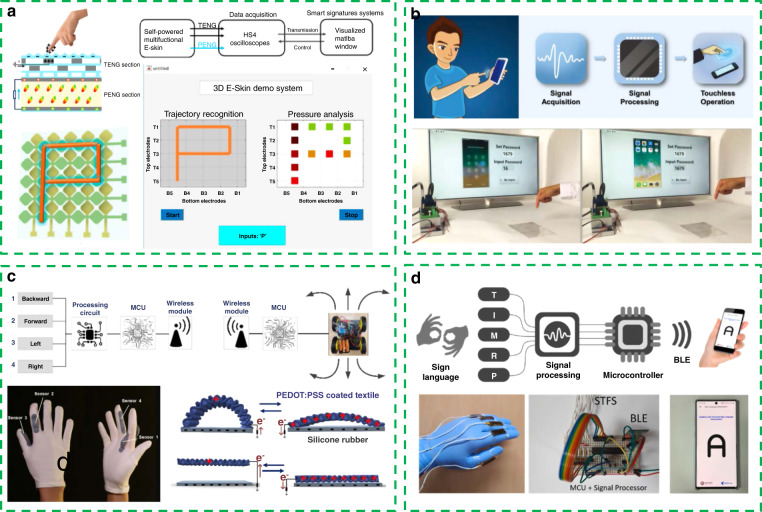
Portable and wearable self-powered systems as human–machine interaction interfaces. **a** Multifunctional electronic skin as a smart anti-counterfeiting signature system (SASS). Reprinted from ref. ^97^ with permission. **b** Triboelectric touch-free screen sensor (TSS). Reprinted from ref. ^98^ with permission. **c** Glove-based multifunctional human–machine interface based on TENG. Reprinted from ref. ^99^ with permission. **d** Self-powered triboelectric flex sensor (STFS) for sign language interpretation. Reprinted from ref. ^100^ with permission.

In Fig. 5a, Guo et al. proposed a multifunctional electronic skin based on the synchronized triboelectrification and piezoelectric effect^97^. Through the design of network cross electrodes, the electronic skin can identify the motion trajectory in a full plane. A smart anti-counterfeiting signature system with the capability of recognizing the writing habits of people is realized and demonstrates its great application potential in a human–machine interaction interface and intelligent recognition.

However, the human–machine interface based on the two-dimensional plane mentioned above can only detect limited kinds of gestures, such as touching and sliding. When using the three-dimensional space as the human–machine interaction interface, we can have an increased freedom of detection. Figure 5b shows an awesome concept of a noncontact human–machine interaction^98^. Here, a triboelectric touch-free screen sensor for recognizing various gestures in a noncontact mode shows the ability to detect multiple gestures, such as the drop and lift of a finger with different speeds, making a fist, opening a palm, and flipping the palm in different directions. In addition, this work shows a noncontact screen control system for smart phones, which presents a new touch-free design concept to develop the next generation of screen sensors.

The human hand is an important channel through which people express information. Through the development of wearable electronic devices that can be attached to the hand, it is possible to use rich hand movements to achieve a variety of interactive functions. Figure 5c shows a glove-based multifunctional human–machine interface based on TENG^99^. By matching the output signals under different gestures with specific functions, this human–machine interface is endowed with various functions, such as wireless car control, wireless drone control, minigame control, and VR game control.

In Fig. 5d, Maharjan et al. developed another self-powered triboelectric flex sensor that can be attached to a finger^100^. Additionally, based on the monitoring of a finger posture, this work shows a real-time application of sign language interpretation. It can be predicted that in the future, there will be more interactive functions beyond imagination, which can be realized through a self-powered human–machine interface.

Artificial intelligence technology represented by machine learning has infused vigor into the development of wearable electronic devices, which enables wearable electronic devices to realize more complex and accurate functions.

**Fig. 6:**
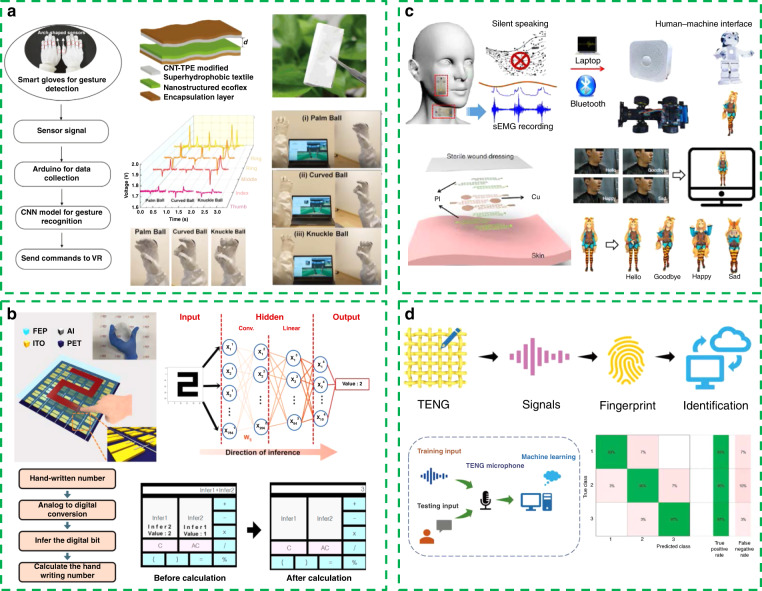
Portable and wearable self-powered systems assisted with machine learning technology. **a** Self-powered machine learning glove based on a superhydrophobic triboelectric fabric structure. Reprinted from ref. ^101^ with permission. **b** Self-powered triboelectricity-based touchpad (TTP). Reprinted from ref. ^102^ with permission. **c** Epidermal sEMG tattoo-like patch for silent speech recognition. Reprinted from ref. ^103^ with permission. **d** TENG-based smart electronics for both voice signal recognition and handwritten signal recognition. Reprinted from ref. ^104^ with permission.

For example, a self-powered machine learning glove based on a superhydrophobic triboelectric fabric structure is proposed in Fig. 6a^101^. With the aid of a machine learning algorithm (convolutional neural network), the glove can achieve very accurate virtual reality/augmented reality (VR/AR) controls, including gun shooting, baseball pitching, and flower arrangement.

The self-powered triboelectricity-based touchpad (TTP) presented in Fig. 6b^102^ has the same basic function as the electronic skin based on the piezoelectric-triboelectric principle^97^. However, the introduction of a pretrained neural network greatly enhances the accuracy and real-time trajectory recognition. Therefore, the TTP can be implemented as the smart calculator by using graphical user interface modeling.

The next generation of the computer revolution is from a graphical interface to a voice user interface, so voice recognition technology is a very competitive development direction in human–machine interaction.

Surface electromyography (sEMG) generated from the jaw contains valuable voice information. Therefore, Liu et al. demonstrated an epidermal sEMG tattoo-like patch with the ability of sEMG recording for silent speech recognition, as shown in Fig. 6c^103^. With the aid of wavelet decomposition and pattern recognition, the average accuracy of action instructions can reach 89.04%, and the average accuracy of emotion instructions is as high as 92.33%. Based on this performance, this tattoo-like patch can even be expected to help aphasia patients regain the ability to communicate with others.

Similarly, the TENG-based smart electronics in Fig. 6d has the ability of both voice signal recognition and handwritten signal recognition^104^. The “medium Gaussian support vector machine” is used as the machine learning model, and a recognition accuracy of 93.5% is obtained.

In summary, the combination of a self-powered system and machine learning technology makes wearable electronic devices more intelligent. It is worth mentioning that in the current work, self-powered intelligent systems mainly possess the two functions of recognition (Figs. 5a, d and 6b, d) and control (Figs. 5b, c and 6a, c), playing the role of assisting humans. In future developments, we can even devote ourselves to empower traditional electronics to “think,” “analyze,” and “decide” and realize the real integration of electronic devices and the human body.

## Summary and perspective

Self-powered systems show great potential in energy harvesting, sensing, actuating, and human–machine interaction applications and are expected to become the main form of electronic devices in the Internet of Things era. In this review, we give a comprehensive introduction to the development of portable and wearable self-powered systems. We started with portable and wearable energy harvesting technology as the basis of self-powered systems and then introduced the application of self-powered systems in the wearable sensing of a series of physical and physiological indicators. In addition, we also introduced typical self-powered systems with actuating functions. Intelligentization is an inevitable trend in the development of electronic equipment. Therefore, we focus on the state-of-the-art developments of portable and wearable self-powered systems as a new type of human–machine interaction interface and demonstrate the importance of machine learning in empowering self-powered systems with higher-level functions. At the same time, we also discussed the application characteristics of different forms of portable wearable self-powered systems in different scenarios.

**Fig. 7:**
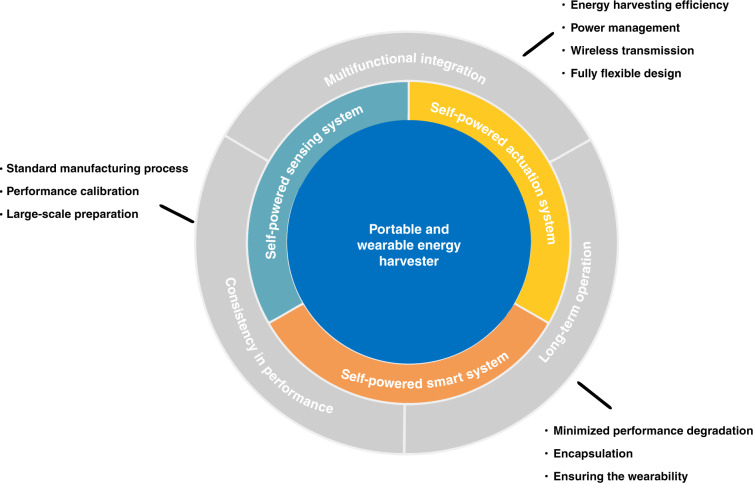
Challenges and prospects of portable and wearable self-powered systems.

However, before the portable and wearable self-powered system can move toward large-scale practical applications, there are still many problems that should be fully addressed in the near future (Fig. 7).

1. Most wearable and portable self-powered systems are based on flexible materials, which experience device performance degradation during long-term operation. Therefore, in the future, we should study additional material and structural designs to improve the stability of the system under long-term work while ensuring the wearability of the device.
2. Currently, portable wearable self-powered electronic devices are mainly desktop laboratory devices, which only demonstrate a concept. Since there is no work to propose a standardized manufacturing process for portable and wearable self-powered electronic devices, it is impossible to achieve complete consistency with respect to the performance of two different devices. Therefore, in the future, we need to consider the issue of performance calibration between different devices, or we can develop standardized processes for portable wearable devices that can be mass produced.
3. Hybrid energy harvesting technology that integrates multiple transduction methods is most likely to act as a power source for future self-powered systems. However, the large differences in the frequency, amplitude, and waveform of electrical power converted through different transduction methods make it an unsolved problem to develop power management technologies suitable for different energy harvesting methods. Furthermore, an increasing number of functional modules are being integrated into self-powered systems. We need to rationally design power management circuits to improve the energy conversion efficiency and achieve energy distribution among various functional modules. In addition, a wireless module is needed to realize the transmission of information.

In summary, portable and wearable self-powered systems have made significant progress and may become the main form of the next generation of electronic devices. The rapid development of materials science, processing technology, and smart technology will pave the way for wearable self-powered systems in practical applications.
